# Corona-Driven Microdroplet Coalescence on an Open Oil Film with Intelligent Detection and Tracking

**DOI:** 10.3390/mi17080878

**Published:** 2026-07-24

**Authors:** Xinyi Qiu, Xiaxia Cui, Yiqing Liu, Hui Liu, Biao Cheng, Jiahan Zhang, Qiang Tang

**Affiliations:** 1 School of Artificial Intelligence, Anhui University of Science and Technology, Huainan 232000, China; 2 State Key Laboratory of Digital Intelligent Technology for Unmanned Coal Mining, Anhui University of Science and Technology, Huainan 232000, China; 3 School of Electronic Information Engineering, Inner Mongolia University, Hohhot 010021, China

**Keywords:** corona discharge, microdroplet coalescence, electrohydrodynamics, YOLOv5, droplet tracking, electro-demulsification

## Abstract

Open-surface coalescence of microdroplets is essential for droplet-based microreactors, emulsion processing, and multiphase microfluidics, yet existing methods often require closed channels or patterned electrodes. Here, we report a corona-driven open-oil-film platform that achieves voltage-regulated coalescence of aqueous microdroplets in a simple needle–plate electrode configuration. Positive corona discharge induces coupled electrohydrodynamic effects—including ion transport, interfacial charge redistribution, and Maxwell stresses—that drive oil-film contraction and charge-regulated droplet bouncing, thereby reducing inter-droplet spacing and promoting successive merging. The coalescence rate and final droplet size are tunable via the applied voltage and oil volume: complete coalescence into a single droplet is achieved at 12 kV, and an optimal oil volume of 60 μL maximizes confinement efficiency. To enable quantitative, frame-by-frame analysis, we develop an improved YOLOv5–OC-SORT framework that yields an overall mAP@0.5 of 0.905 for automatic droplet detection and tracking. As a proof-of-concept, the platform achieves electro-demulsification of a surfactant-stabilized water-in-oil emulsion, increasing the average droplet diameter from ~0.005 mm to ~0.2 mm and enabling effective oil–water separation. This work provides a simple, electrode-pattern-free strategy for controllable droplet coalescence and open-surface emulsion breaking.

## 1. Introduction

Controlled microdroplet coalescence is central to multiphase fluid systems, with broad relevance to droplet-based microreactors, biochemical assays, emulsion processing, oil–water separation, and open-surface microfluidics [1,2]. Regulated merging enhances interfacial mass transfer, accelerates chemical reactions, and enables phase separation. For water-in-oil emulsions, the coalescence of dispersed aqueous droplets is a prerequisite for demulsification [3,4]. Yet, microscale droplets in an oil phase are inherently stabilized by interfacial tension, viscous resistance, and surfactant adsorption, all of which suppress droplet contact and hinder drainage and rupture of the intervening oil film [5,6]. Consequently, spontaneous coalescence is typically slow, inefficient, and poorly controllable.

To overcome these limitations, various external-field and microfluidic strategies have been developed, including microchannel confinement, electric-field actuation, acoustic excitation, optical heating, magnetic manipulation, and mechanical agitation [7,8,9,10]. While these methods can enhance collision or destabilize the intervening film, they typically require closed channels, patterned electrodes, complex chip architectures, or direct mechanical disturbance [9,11,12]. Such constraints limit their adaptability for open-surface operation, large-area manipulation, and simple emulsion treatment. Conventional electrocoalescence, for instance, requires electrodes in direct contact with or in close proximity to the liquid, while microfluidic approaches are constrained by channel dimensions and fabrication complexity [13,14,15]. In surfactant-stabilized emulsions, coalescence is further inhibited by the adsorbed interfacial layer, which resists droplet deformation and retards drainage and rupture of the intervening oil film. Effective demulsification therefore requires not only enhanced droplet collision but also electrical or hydrodynamic destabilization of the surfactant-covered interface [16].

Contactless corona-based methods have recently been investigated for electrocoalescence [17], droplet oscillation [18], dielectric-liquid transport [19], and oil–water separation [20]. In these systems, ions generated by a needle electrode induce charge injection, interfacial electrical stresses, and electrohydrodynamic motion without direct contact between the high-voltage electrode and the liquid [21]. However, previous studies have focused primarily on individual-droplet actuation, bulk emulsion treatment, or transport along predefined conductive pathways. By contrast, the controllable collective coalescence of a dispersed microdroplet population on an open oil film remains insufficiently explored. Accordingly, a simple and scalable strategy that does not require microscale patterned electrode arrays is still needed for open-surface droplet coalescence.

Here, we develop a corona-driven open-oil-film platform for voltage-regulated collective coalescence of aqueous microdroplets. Unlike previous corona-based studies focused primarily on individual-droplet actuation, phase-selective transport, or bulk emulsion treatment, the present platform exploits the coupled effects of corona-induced oil-film contraction and charge-regulated droplet motion to confine a dispersed microdroplet population and promote successive many-droplet coalescence on an open surface. We systematically quantify the effects of applied voltage and oil volume on the coalescence dynamics and final coalescence degree and introduce an improved YOLOv5–OC-SORT framework for automated frame-by-frame droplet detection and tracking. Finally, the same platform is applied to a surfactant-stabilized water-in-oil emulsion to demonstrate coalescence-assisted demulsification. This integration of dynamically generated oil-film confinement, tunable collective coalescence, and intelligent quantitative analysis constitutes the principal technological advance of this work.

## 2. Results and Discussion

**Figure 1:**
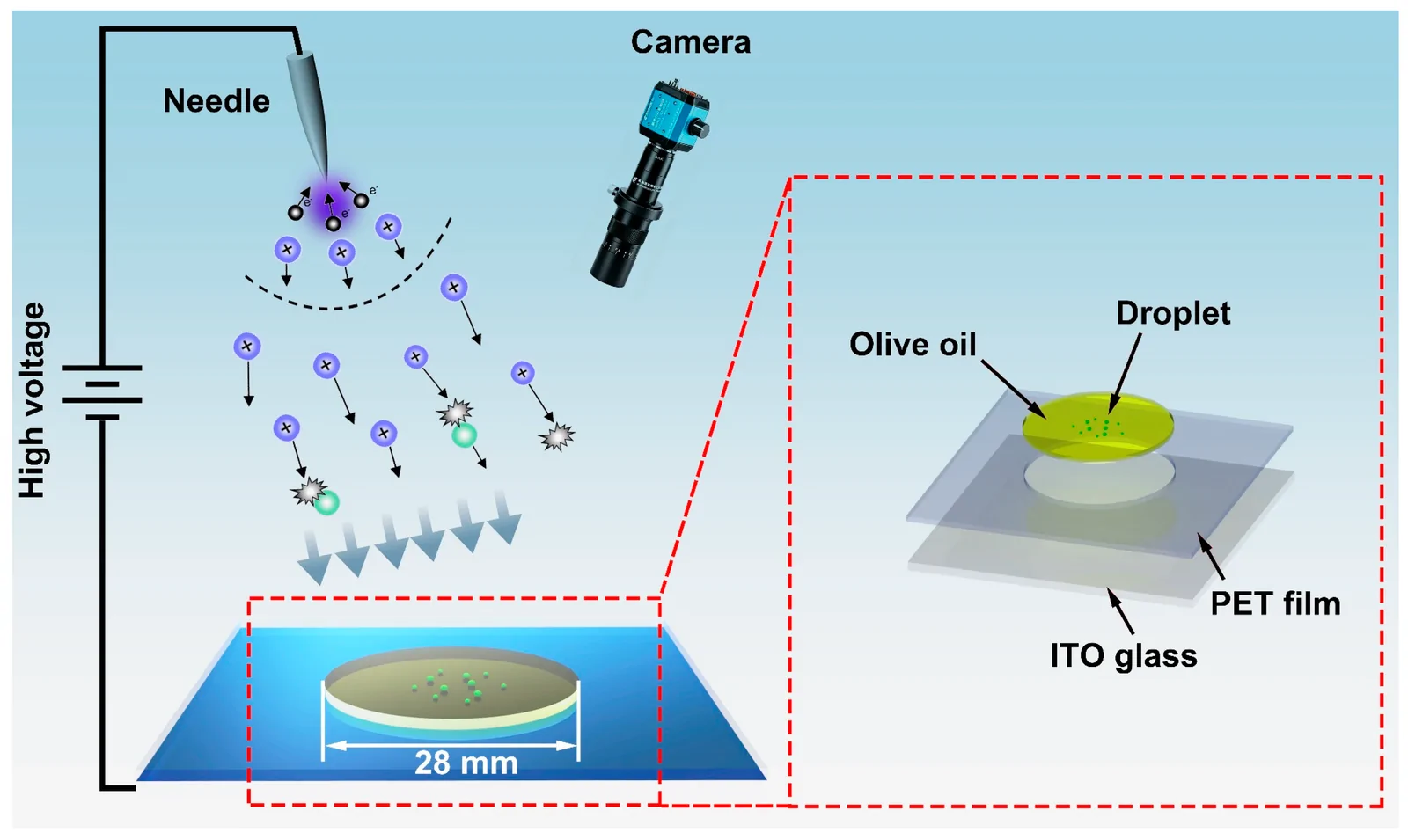
Experimental setup and working principle of the corona-driven open-oil-film microdroplet coalescence system.

As shown in Figure 1, the experimental system consists of a needle–plate electrode configuration powered by a high-voltage direct current (DC) power supply (Dongwen Co., DW-P303-5ACCC, Dalian, China). The needle electrode, with a cone angle of 30°, a tip curvature radius of 2 μm, and a diameter of 350 μm, was connected to the positive terminal of the power supply. An indium tin oxide glass plate (ITO glass, Xiangcheng & Technology, STN, Yiyang, China; 100 × 100 × 0.5 mm^3^) was used as the plate electrode and connected to the negative terminal. The vertical distance between the needle tip and the plate electrode was fixed at 3.5 cm. The ITO surface was covered with a 60 μm-thick PET film with a surface roughness of 1.76 nm (Huajiu, B-55, Hangzhou, China). A circular conductive working region with a diameter of 2.5 cm was patterned at the center of the PET film to accommodate the oil layer and aqueous droplets, and the needle tip was aligned directly above the center of this region. Olive oil was selected as the dielectric liquid owing to its high electrical resistance, thermal and chemical stability, low cost, and wide availability. Dyed deionized water was used as the aqueous droplet phase. During the experiment, a defined volume of olive oil was first deposited onto the circular working region using a pipette. Subsequently, microscale dyed deionized water droplets were generated and deposited onto the oil layer by ultrasonic atomization. The atomization time was fixed at 1 min for each experiment to ensure comparable initial droplet distributions. After droplet deposition, the high-voltage power supply was turned on to initiate corona discharge at the needle tip. The discharge released ions toward the oil surface, thereby triggering oil-film deformation, droplet oscillation, and voltage-regulated droplet coalescence.

**Figure 2:**
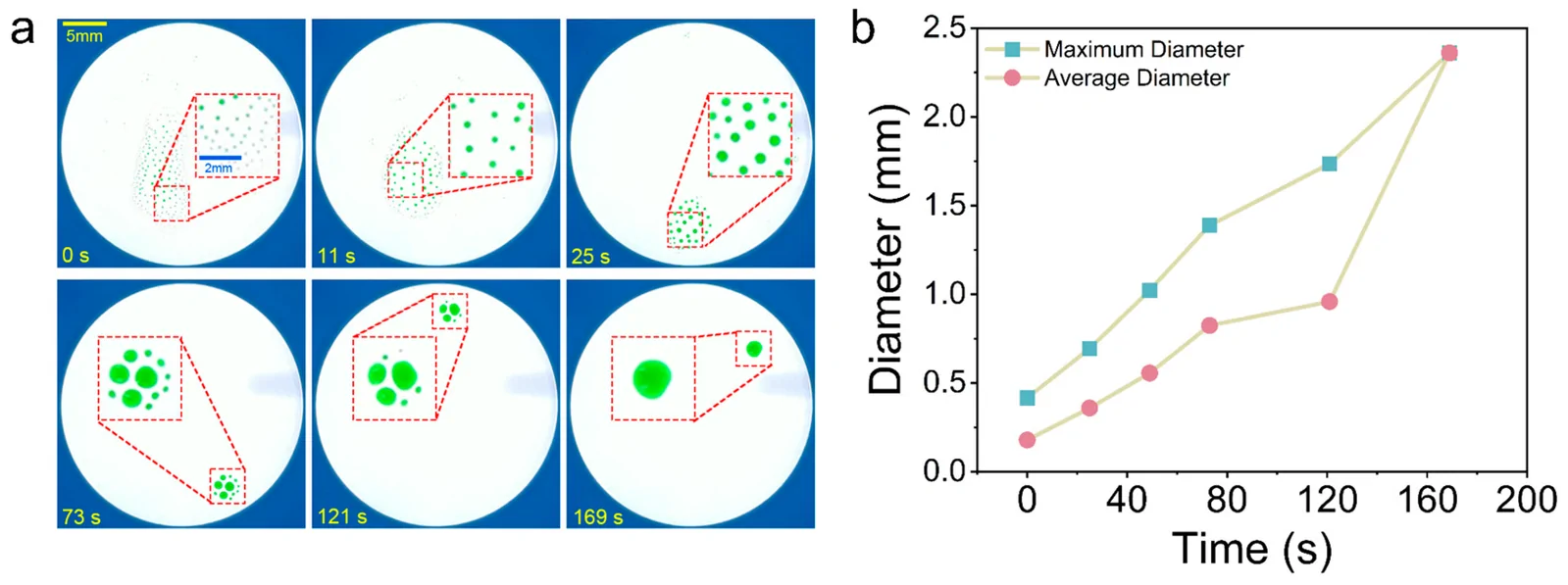
Time-resolved coalescence behavior of dyed water microdroplets on an open oil film. (**a**) Time-lapse images showing the coalescence process of dyed deionized water microdroplets deposited on the olive oil film (Movie S1). In (**a**), the green dots represent dyed aqueous droplets, and the red dashed boxes and connecting lines indicate the selected regions and their corresponding enlarged views. (**b**) Temporal evolution of the maximum droplet diameter and average droplet diameter during the coalescence process.

Figure 2a shows the time-resolved evolution of dyed aqueous microdroplets deposited on the oil film at an oil volume of 80 μL and an applied voltage of 12 kV. Initially, numerous small droplets were randomly dispersed across the olive-oil film. Upon activation of the corona discharge, the oil layer underwent electrohydrodynamically induced deformation and contraction. This contraction progressively confined the dispersed droplets, reduced the inter-droplet spacing, and increased the probability of droplet–droplet collisions. As the confinement intensified, successive coalescence events transformed the initially dispersed droplet population into fewer and larger droplets, ultimately resulting in complete merging into a single droplet. The corona-discharge current was monitored using a microammeter connected in series with the high-voltage circuit. Under a representative operating condition of 12 kV and 60 μL of oil, the measured current was approximately 50 μA, corresponding to an electrical power consumption of approximately 0.6 W, as calculated from (P = VI).

The quantitative variation in droplet size is shown in Figure 2b. The maximum droplet diameter increased continuously from 0.417 mm at 0 s to 2.360 mm at 169 s, while the average droplet diameter increased from 0.179 mm to 2.360 mm over the same period. This monotonic increase in both the maximum and average diameters confirms the progressive coalescence of microdroplets. In particular, the gradual growth of the average diameter indicates that small droplets were continuously consumed through merging events, whereas the increase in the maximum diameter reflects the formation and growth of dominant larger droplets. At 169 s, the maximum and average diameters became identical, indicating that the dispersed droplet population had completely coalesced into a single final droplet. These results demonstrate that the corona-driven oil-film confinement can effectively promote and regulate the coalescence of microscale aqueous droplets on an open oil interface.

**Figure 3:**
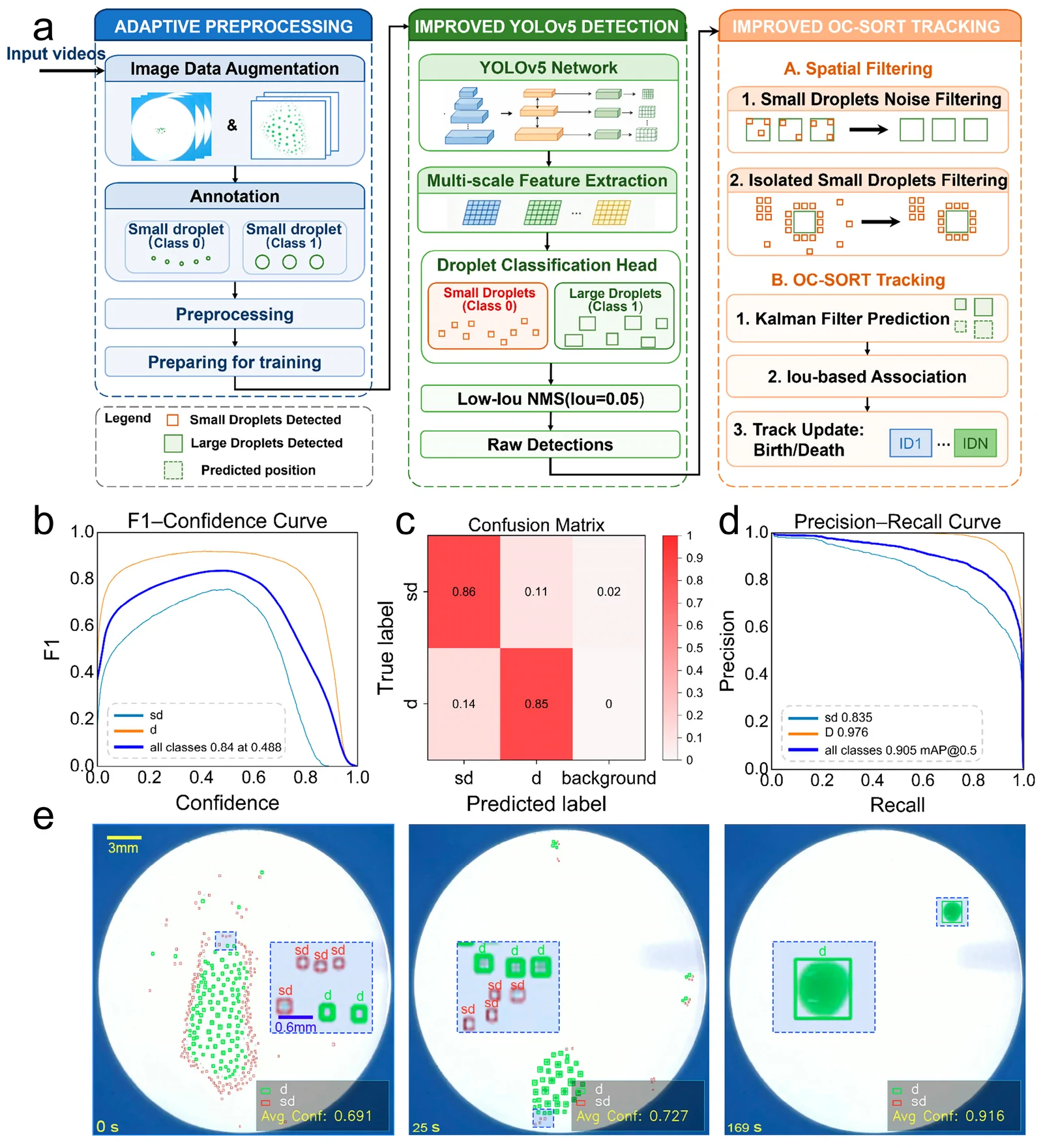
AI-assisted detection and tracking framework for quantitative analysis of droplet coalescence. (**a**) Schematic workflow of the improved YOLOv5–OC-SORT framework for droplet detection and tracking. The framework consists of three modules: adaptive preprocessing, improved YOLOv5 detection, and improved OC-SORT tracking. (**b**) F1-confidence curves for small droplets, large droplets, and all classes. The optimal confidence threshold for all classes was 0.488, corresponding to an F1 score of 0.84. (**c**) Normalized confusion matrix of the trained detector. The recall values for small droplets and large droplets were 0.86 and 0.85, respectively. (**d**) Precision–recall curves of the detector. The AP@0.5 values for small droplets and large droplets were 0.835 and 0.976, respectively, with an overall mAP@0.5 of 0.905. (**e**) Representative detection and tracking results during the droplet coalescence process at 0, 25, and 169 s. Small droplets are marked as sd, and large droplets are marked as d (Movie S2).

Droplet recognition and size quantification were initially performed manually, which was time-consuming and prone to subjective error. To enable automatic and frame-by-frame analysis, an improved YOLOv5–OC-SORT framework was developed for droplet detection and tracking, as shown in Figure 3a. The framework classified droplets into two categories: small droplets before coalescence, denoted as class 0 or sd, and large droplets formed after merging, denoted as class 1 or d. In this work, droplets with a diameter greater than 0.2 mm were defined as large droplets.

The framework consisted of three main modules. First, adaptive preprocessing was used to improve data quality, including image augmentation, droplet annotation, black-border cropping, and optional CLAHE enhancement and sharpening. These steps reduced irrelevant background interference and enhanced the visibility of small, low-contrast droplets. Second, YOLOv5s was adopted as the baseline detector to identify droplets of different sizes through multi-scale feature extraction. A two-class detection head was used to distinguish small and large droplets, and low-IoU non-maximum suppression with an IoU threshold of 0.05 was applied to remove duplicated detections. Third, an improved OC-SORT tracking module was used to associate droplets across consecutive frames. Spatial filtering was first introduced to remove false small-droplet detections inside large droplets and isolated noise-like detections. Then, Kalman-filter prediction, IoU-based association, velocity-direction consistency checking, and track life-cycle management were combined to assign each droplet a stable tracking ID. Detailed training parameters and implementation procedures are provided in Supplementary Note S1.

The detection performance is summarized in Figure 3b–d. The F1-confidence curve in Figure 3b shows that the optimal confidence threshold was 0.488, corresponding to an F1 score of 0.84. The normalized confusion matrix in Figure 3c gives recall values of 0.86 and 0.85 for small and large droplets, respectively. The main error source was the misclassification between small and large droplets. As shown in Figure 3d, the AP@0.5 values for small and large droplets were 0.835 and 0.976, respectively, with an overall mAP@0.5 of 0.905. Representative detection and tracking results are shown in Figure 3e. At 0 s, many small droplets were distributed on the oil film and were successfully detected as sd. At 25 s, partial coalescence had occurred, and the model simultaneously recognized residual small droplets and newly formed large droplets. At 169 s, the droplets had almost completely coalesced into a final large droplet, which was correctly identified as d. These results confirm that the improved YOLOv5–OC-SORT framework can reliably quantify droplet number, size evolution, trajectories, and coalescence degree during corona-driven microdroplet coalescence.

**Figure 4:**
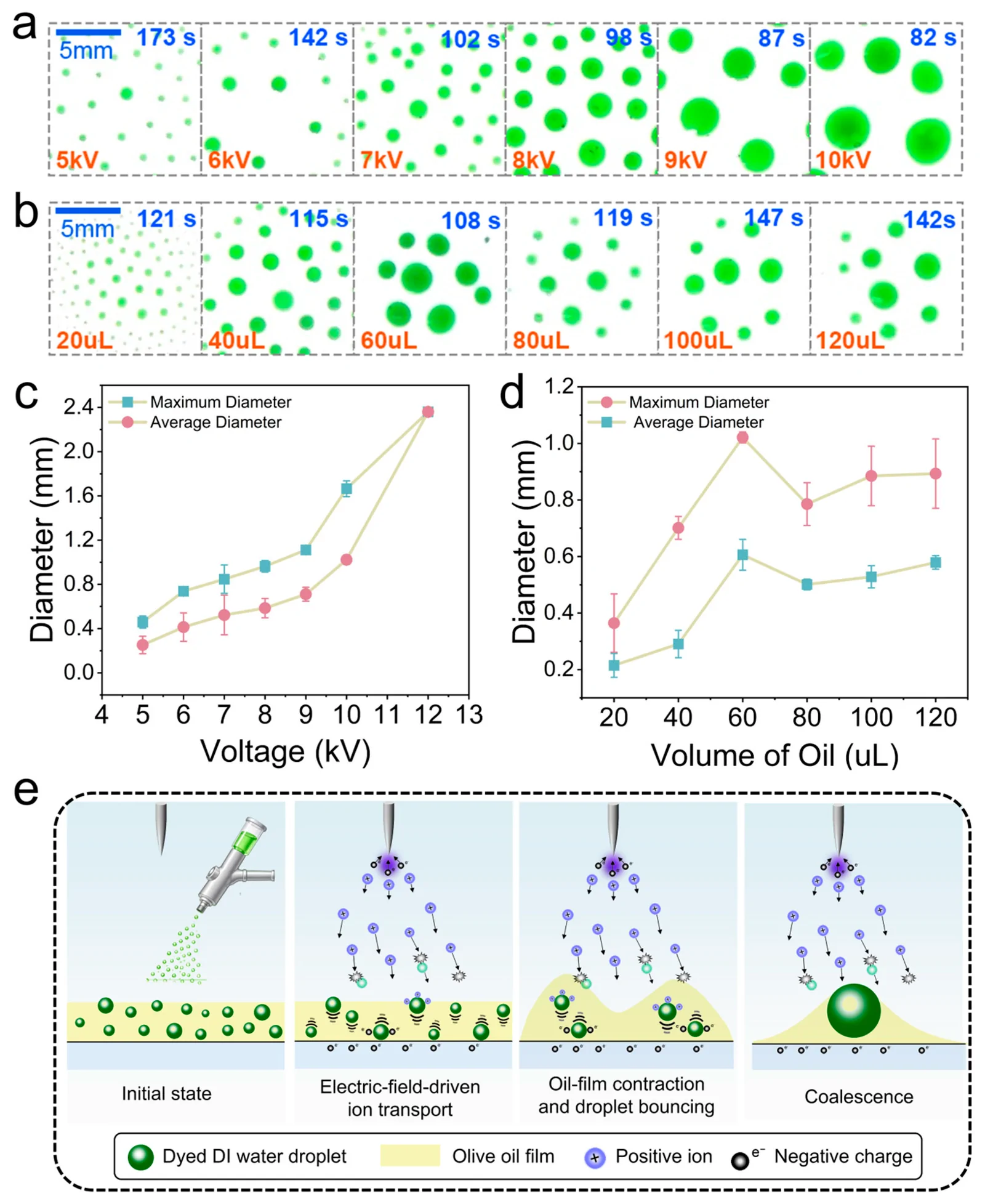
Effects of applied voltage and oil volume on corona-driven microdroplet coalescence. (**a**) Representative final-state images of droplet coalescence under different applied voltages from 5 to 10 kV at a fixed oil volume of 60 μL. (**b**) Representative final-state images of droplet coalescence under different oil volumes at a fixed applied voltage of 8 kV. (**c**) Maximum droplet diameter and average droplet diameter as functions of applied voltage. (**d**) Maximum droplet diameter and average droplet diameter as functions of oil volume. (**e**) Schematic illustration of the proposed coalescence mechanism.

To evaluate the controllability of corona-driven droplet coalescence, the effects of applied voltage and oil volume were investigated. Figure 4a shows representative final-state images obtained at different voltages from 5 to 10 kV with a fixed oil volume of 60 μL. The time labeled in each image represents the stabilization time, at which no further obvious coalescence occurred. At low voltages, the weak electric-field strength and corona-induced interfacial forcing resulted in limited oil-film contraction and incomplete droplet merging. As the voltage increased, stronger corona discharge enhanced ion transport, interfacial charge accumulation, and electrohydrodynamic flow, leading to faster oil-film contraction, more frequent droplet collisions, and more complete coalescence.

The quantitative results in Figure 4c further confirm this voltage dependence. As the voltage increased from 5 to 12 kV, the maximum droplet diameter increased from 0.459 mm to 2.360 mm, while the average diameter increased from 0.252 mm to 2.360 mm. At 12 kV, the maximum and average diameters became identical, indicating complete coalescence into a single droplet. These results demonstrate that the applied voltage can effectively regulate both the coalescence rate and final coalescence degree.

Oil volume also had a pronounced effect on droplet coalescence. Figure 4b shows the final states obtained at different oil volumes under a fixed applied voltage of 8 kV. At low oil volumes, the oil layer provided insufficient continuity, deformability, and interfacial mobility, resulting in weak corona-induced contraction, limited droplet transport, and poor coalescence. As the oil volume increased, film continuity and electrohydrodynamic contraction were enhanced, with the best coalescence performance observed at 60 μL. As shown in Figure 4d, the maximum droplet diameter increased from 0.364 mm at 20 μL to 1.021 mm at 60 μL, while the average diameter increased from 0.215 to 0.606 mm. This nonmonotonic dependence can be understood as a balance among oil-film continuity, lateral confinement, droplet mobility, and drainage of the intervening oil film. As reported for confined droplet coalescence in microchannels, successful merging requires both sufficient droplet approach and subsequent drainage and rupture of the continuous-phase film between neighboring droplets [22]. At 60 μL, the oil layer remained sufficiently continuous and deformable while maintaining effective lateral confinement, thereby reducing inter-droplet spacing and facilitating collision and film drainage. Further increases in oil volume led to a decrease in final droplet size followed by a plateau, likely because the thicker and more extended oil layer weakened confinement, increased droplet transport distances and bulk hydrodynamic dissipation, and potentially prolonged drainage of the intervening oil film. Therefore, the optimum observed at 60 μL under 8 kV reflects a favorable balance between film continuity, confinement, and drainage dynamics.

The proposed mechanism is illustrated in Figure 4e. Initially, numerous dyed aqueous microdroplets are dispersed on the olive oil film. After a positive voltage is applied to the needle electrode, corona discharge generates ions that migrate toward the negatively biased ITO plate electrode. Ion accumulation and relaxation near the oil–air interface produce nonuniform interfacial charges and Maxwell stresses, which deform the oil film and induce electrohydrodynamic contraction. Meanwhile, aqueous droplets are polarized and acquire transient charges through capacitive coupling and interaction with corona-injected ions, leading to repeated bouncing within the oil layer. Oil-film contraction reduces inter-droplet spacing, while droplet bouncing increases collision frequency. Once neighboring droplets contact each other, electrostatic attraction and collision-induced drainage of the intervening oil film promote coalescence.

To identify the dominant driving forces governing the voltage- and oil-volume-dependent coalescence, we performed an order-of-magnitude force balance analysis considering Maxwell stress, interfacial tension, viscous drag, and electrostatic attraction between droplets. At 12 kV, the electric capillary number $Ca_{E}=\varepsilon_{0}\varepsilon_{\mathrm{oil}}E^{2}R/\gamma \approx 1.10>1$, confirming that Maxwell stress overcomes surface tension to drive droplet deformation and coalescence. In contrast, viscous drag ($F_{\mu }∼10^{-8}$ N) is approximately three orders of magnitude smaller than the electric force ($F_{E}∼10^{-5}$ N), indicating that viscous dissipation is not the primary resistive mechanism. The complete derivation and detailed force estimates are provided in Supplementary Note S2 and Table S1. Overall, corona-driven coalescence is governed by the coupled effects of voltage-dependent electrical forcing and oil-volume-dependent confinement, enabling tunable droplet merging on an open oil-film interface.

To further distinguish the role of corona-induced ion transport from that of the applied electric field alone, a control experiment was performed using an insulated needle tip (Pure-field tip) that suppresses corona discharge while maintaining a similar electric field distribution. Under identical conditions (8 kV, 60 μL oil), the bare needle tip (Corona tip) induced significant droplet migration and coalescence, whereas the insulated tip produced almost no observable droplet motion (Supplementary Figure S2 and Movies S4 and S5). This control experiment unambiguously demonstrates that the applied electric field alone is insufficient to drive droplet coalescence, and that the ion transport generated by corona discharge plays an essential and irreplaceable role.

**Figure 5:**
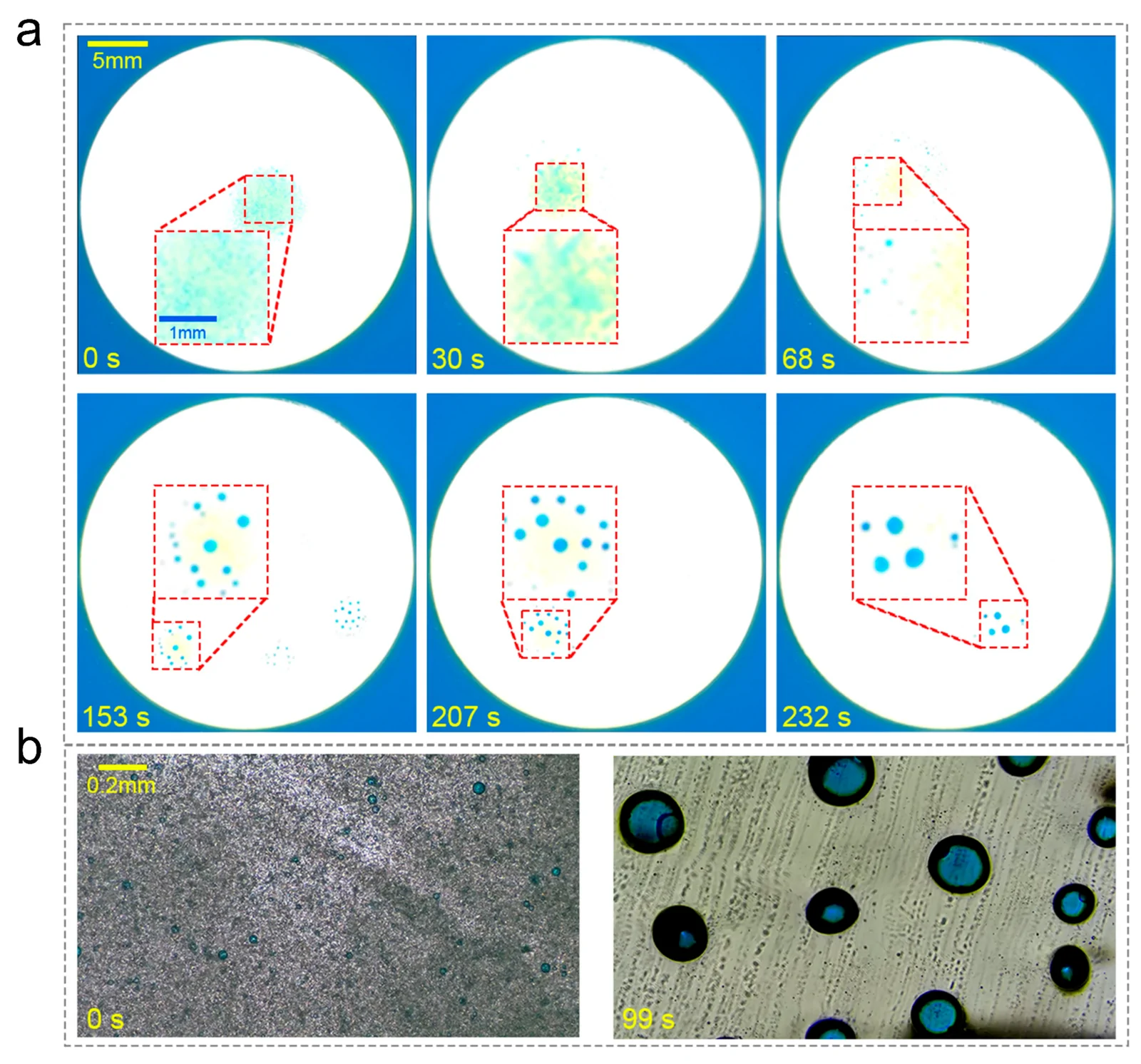
Application of corona-driven droplet coalescence for electro-demulsification and oil–water separation. (**a**) Macroscopic demonstration of the demulsification of a water-in-oil emulsion under corona discharge. The blue/cyan dots represent dyed aqueous droplets, and variations in apparent color intensity arise from differences in droplet size and imaging conditions. The red dashed boxes and connecting lines indicate the selected regions and their corresponding magnified views. (**b**) Microscopic observation of the demulsification process (Movie S3).

To demonstrate the application potential of the corona-driven coalescence strategy, the system was applied to electro-demulsification and oil–water separation. A model water-in-oil emulsion was prepared using linseed oil as the continuous phase and 1 wt% SDS aqueous solution as the dispersed phase, with a water-to-oil volume ratio of 2:8. The two phases were ultrasonically emulsified to form a stable emulsion. During demulsification, the needle-to-plate distance was fixed at 3.5 cm, and an applied voltage of 8 kV was used to initiate corona discharge. As shown in Figure 5a, the freshly prepared emulsion was macroscopically uniform before electrical treatment. After corona discharge was applied, the emulsion gradually became unstable: dispersed water droplets collided, coalesced into larger droplets, and finally separated from the oil phase, enabling oil–water separation. Microscopic observations in Figure 5b further reveal the dynamic demulsification process. Initially, small water droplets were densely dispersed in the oil phase, with an average diameter of approximately 0.005 mm. Under corona discharge, the droplets exhibited obvious vibration and oscillatory motion, while the surrounding oil phase showed visible local flow. These motions promoted droplet transport, collision, and rapid coalescence. After treatment, the average droplet diameter increased to approximately 0.2 mm, corresponding to a 40-fold increase in characteristic droplet size.

The demulsification process is attributed to the coupling of droplet polarization, corona-induced charge transport, and electrohydrodynamic oil-phase motion. The nonuniform electric field induces transient charging and vibration of dispersed water droplets, while interfacial electrical stresses drive local oil flow and enhance droplet contact. Repeated collision and deformation accelerate drainage and rupture of the thin oil film between adjacent droplets, leading to coalescence. Quantitative benchmarking against existing technologies (Supplementary Table S2) shows that our platform achieves energy consumption (0.3–3.3 kWh/m^3^) comparable to state-of-the-art electric demulsification membranes [23] (3 kWh/m^3^) and centrifugation [24] (1.0–4.0 kWh/m^3^), while offering the unique combination of non-contact operation, chemical-free processing, and real-time accessibility.

## 3. Conclusions

In this work, we developed a corona-driven open-oil-film platform for controllable collective coalescence of randomly dispersed aqueous microdroplets. By coupling corona-induced electrical forcing with dynamic oil-film contraction, the system creates a self-generated lateral confinement effect that reduces inter-droplet spacing, enhances droplet mobility, and promotes successive coalescence on an open surface. The coalescence behavior was regulated by the applied voltage and oil volume, with complete merging into a single millimeter-scale droplet achieved at 12 kV and the best performance observed at 60 μL under 8 kV. An improved YOLOv5–OC-SORT framework enabled automated frame-by-frame detection and tracking with an overall mAP@0.5 of 0.905. The platform also facilitated the breaking of a surfactant-stabilized water-in-oil emulsion, increasing the average droplet diameter from approximately 0.005 to 0.2 mm and promoting visible phase separation. The principal contribution of this work is therefore the use of dynamically generated oil-film confinement to overcome the low collision probability of dispersed droplets in an open system, providing a simple strategy for open-surface droplet manipulation, quantitative coalescence analysis, and emulsion treatment.

## Data Availability

The original contributions presented in this study are included in the article/Supplementary Material. Further inquiries can be directed to the corresponding authors.

## Supplementary Materials

The following supporting information can be downloaded at: https://www.mdpi.com/article/10.3390/mi17080878/s1, Supplementary Note S1: YOLOv5–OC-SORT model training and tracking implementation; Supplementary Note S2: Quantitative force balance analysis; Figure S1: Quantitative characterization of the initial experimental conditions; Figure S2: Experimental evidence for corona-induced oil-film contraction, droplet bouncing, and ion-transport-mediated coalescence; Table S1: Order-of-magnitude force balance analysis under typical experimental conditions (12 kV, 60 μL oil, *R* = 0.1 mm); Table S2: Quantitative comparison of oil–water separation technologies; Movie S1: The coalescence process of dyed deionized water microdroplets deposited on the olive oil film; Movie S2: AI-assisted detection and tracking of corona-driven droplet coalescence; Movie S3: Macroscopic and microscopic visualization of corona-driven electro-demulsification; Movie S4: Corona-induced oil-film contraction and charge-related droplet bouncing; Movie S5: Control experiments distinguishing corona-ion transport from the pure electric-field effect.
