# Supplementary material for: Corona-Driven Microdroplet Coalescence on an Open Oil Film with Intelligent Detection and Tracking
Source: Micromachines (Basel). 2026 Jul 24;17(8):878. doi: 10.3390/mi17080878 (PMC13515563; doi:10.3390/mi17080878)
Supplement: Supplementary file 1 [file micromachines-17-00878-s001.zip › Support information.pdf]

Supporting Information

Corona-Driven Microdroplet Coalescence on an Open Oil Film with Intelligent Detection and Tracking

Xinyi Qiu [1], Xiaxia Cui [1,2,*], Yiqing Liu [1], Hui Liu [1], Biao Cheng [1], Jiahan Zhang [3] and Qiang Tang [1,2,*]

1 School of Artificial Intelligence, Anhui University of Science and Technology, Huainan 232000, China

2 State Key Laboratory of Digital Intelligent Technology for Unmanned Coal Mining, Anhui University of Science and Technology, Huainan 232000, China

3 School of Electronic Information Engineering, Inner Mongolia University, Hohhot 010021, China

* Correspondence: xiaxiacui@whu.edu.cn (X.C.); tangqiang@whu.edu.cn (Q.T.)

This PDF file includes:

Note S1 to S2

Table S1 to S2

Legends for Movies S1 to S5

Other supplementary materials for this manuscript include the following:

Movies S1 to S5

Supplementary Note S1: YOLOv5–OC-SORT model training and tracking implementation

For model training, an initial dataset containing 196 images was first manually annotated and used to train a preliminary YOLOv5 model. The preliminary model was then integrated with X-AnyLabeling to perform AI-assisted batch pre-annotation. After manual correction and verification, the dataset was expanded to 1000 annotated images. The dataset was divided into training and validation sets at a ratio of 8:2. Two droplet classes were defined: small droplets (class 0, sd) and large droplets (class 1, d), and a standard YAML configuration file was generated for YOLO training.

YOLOv5s was selected as the base model, and COCO-pretrained weights were used for initialization. The input image size was set to 960 × 960 pixels. The model was trained for 150 epochs with a batch size of 6 using the SGD optimizer. The initial learning rate was 0.005 and was gradually reduced using cosine learning-rate decay. Early stopping was applied if the validation performance did not improve for 50 consecutive epochs. Data augmentation strategies, including Mosaic augmentation, HSV perturbation, horizontal flipping, and random rotation within ±5°, were used to reduce overfitting and improve the robustness of the model. The model with the best validation performance was saved as best.pt and used for subsequent video analysis.

The detection and tracking system was implemented using Python, PyTorch, and OpenCV. Each input frame was first resized and padded to the model input size using the letterbox() operation. The trained YOLOv5 model was then used for forward inference, followed by non-maximum suppression with a confidence threshold of 0.1 and an IoU threshold of 0.05. Detected droplets were separated by class and further processed using spatial filtering. False small-droplet detections inside large droplets were removed, and isolated noise-like detections were filtered based on a neighbor-distance threshold of 60 pixels and a minimum neighbor number of 3. OC-SORT tracking was then performed using an eight-dimensional Kalman-filter state. The predicted tracks and current detections were associated using IoU-based matching and the Hungarian algorithm, with an additional velocity-direction consistency constraint using a cosine similarity threshold of 0.3. Tracks lost for no more than 3 frames were

recovered by secondary IoU matching, and track age and maximum age were used to manage the full life cycle of each droplet trajectory. The final output included the droplet ID, bounding box, and category for each frame, where large droplets were marked with green bounding boxes and small droplets were marked with red center points for subsequent quantitative analysis.

In addition, the initial experimental conditions were systematically characterized to ensure the reproducibility of the subsequent detection, tracking, and coalescence analyses, including the oil-film thickness uniformity, batch-to-batch variation in the droplet population, and initial droplet-size distribution, as shown in Figure S1.

Supplementary Note S2. Quantitative Force Balance Analysis

To quantitatively elucidate the dominant driving forces governing the corona-driven coalescence, we performed an order-of-magnitude force balance analysis considering four primary forces: **Maxwell stress** (F_E), **interfacial tension** (F_γ), **viscous drag** (F_μ), and **electrostatic attraction between droplets** (F_{es}). The analysis was conducted under typical experimental conditions (V=12 kV, oil volume = 60 μL, droplet radius R=0.1 mm). The key parameters are summarized in Supplementary Table S2.

(1) Maxwell stress (F_E) – the primary driving force for oil-film deformation and droplet stretching

The electric stress acting on the oil–air and oil–water interfaces is given by the Maxwell stress tensor:

$$\mathbf{M}_i = \varepsilon_0 \varepsilon_i [\mathbf{E}_i \mathbf{E}_i - \frac{1}{2}(\mathbf{E}_i \cdot \mathbf{E}_i)\mathbf{I}]$$

The normal component of the Maxwell stress produces an electric pressure at the interface:

$$p_E = \frac{1}{2}\varepsilon_0 \varepsilon_{\text{oil}} E_n^2$$

where E_n is the normal electric field. For our needle–plate geometry at $V =$ 12 kV and a gap of 3.5 cm, the average field is $E_{\text{avg}} \approx V/d \approx 3.4 \times 10^5$ V/m. Owing to the sharp needle tip (radius ~2 μm), the local field enhancement factor β is estimated to be of the order $10^2 – 10^3$. Taking a conservative local field $E_{\text{local}} \sim 10^6$ V/m and $\varepsilon_{\text{oil}} \approx 3.1$ (olive oil), we obtain:

$$p_E \approx \frac{1}{2} \times (8.85 \times 10^{-12}) \times 3.1 \times (10^6)^2 \approx 13.7 \text{ Pa}$$

At higher local fields ($E_{\text{local}} \sim 10^7$ V/m), p_E can reach $\sim 10^3$ Pa. The corresponding electric force on a droplet of radius $R \sim 0.1$ mm is:

$$F_E \sim p_E \cdot \pi R^2 \approx 10^3 \times \pi \times (10^{-4})^2 \approx 3.1 \times 10^{-5} \text{ N}$$

This force scales as $F_E \propto V^2$, which explains the strong voltage dependence observed in Figure 4c.

(2) Interfacial tension (F_γ) – the primary resisting force

The capillary pressure opposing droplet deformation is given by the Laplace equation:

$$p_\gamma = \frac{2\gamma}{R}$$

For the olive oil–water interface, $\gamma \approx 0.025$ N/m. For $R = 0.1$ mm:

$$p_\gamma = \frac{2 \times 0.025}{1 \times 10^{-4}} = 500 \text{ Pa}$$

Thus,

$$F_\gamma \sim p_\gamma \cdot \pi R^2 \approx 500 \times \pi \times (10^{-4})^2 \approx 1.57 \times 10^{-5} \text{ N}$$

The **electric capillary number** compares electric stress to surface tension:

$$Ca_E = \frac{\varepsilon_0 \varepsilon_{\text{oil}} E^2 R}{\gamma}$$

At $E \sim 10^6$ V/m and $R = 10^{-4}$ m:

$$Ca_E = \frac{(8.85 \times 10^{-12}) \times 3.1 \times (10^6)^2 \times 10^{-4}}{0.025} \approx 1.10$$

When $Ca_E > 1$, electric stress overcomes surface tension, enabling droplet deformation and stretching. This explains why complete coalescence into a single droplet is achieved at 12 kV ($Ca_E \gg 1$), while at lower voltages ($Ca_E < 1$) coalescence remains incomplete.

(3) Viscous drag (F_μ) – the dominant energy-dissipation mechanism

As droplets move and the oil film contracts, viscous dissipation in the oil phase resists motion. The viscous stress is estimated as:

$$\tau_\mu \sim \mu_{\text{oil}} \frac{U}{h}$$

where $\mu_{\text{oil}} \approx 0.084$ Pa·s (olive oil at 25 °C), UU is the characteristic droplet velocity (estimated from time-lapse imaging as $\sim$ 0.5 mm/s), and hh is the oil film thickness. For 60 μL oil spread over a circular area of diameter 2.5 cm, $h = V/(\pi r^2) \approx 122$ μm. The viscous force on a droplet is:

$$F_\mu \sim \tau_\mu \cdot \pi R^2 = \mu_{\text{oil}} \frac{U}{h} \pi R^2$$

Substituting the values:

$$F_\mu \sim 0.084 \times \frac{5 \times 10^{-4}}{1.22 \times 10^{-4}} \times \pi \times (10^{-4})^2 \approx 1.08 \times 10^{-8} \text{ N}$$

This is approximately **three orders of magnitude smaller** than F_E and F_γ, indicating that viscous drag is not the primary resistance to coalescence under our experimental conditions. However, when the oil volume increases to 80 μL ($h \approx 163$ μm), the thicker film reduces the velocity gradient (U/h decreases), but the increased oil volume also enhances **bulk viscous dissipation** during large-scale oil-film contraction. This increased dissipation partially explains the reduced coalescence efficiency at excessive oil volumes (Figure 4d).

(4) Electrostatic attraction between droplets (F_{es}) – the driving force for droplet–droplet approach

Under the non-uniform electric field, aqueous droplets become polarized and acquire induced dipoles. The dipole–dipole interaction force between two droplets of radius R separated by centre-to-centre distance s can be estimated as:

$$F_{es} \sim \frac{1}{4\pi\varepsilon_0\varepsilon_{\text{oil}}}\frac{p_1 p_2}{s^4}$$

where the induced dipole moment $p \propto \varepsilon_0\varepsilon_{\text{oil}}ER^3$. For two droplets with $R =$ 0.1 mm separated by $s \sim$ 0.5 mm, and taking $E \sim 10^6$ V/m, we obtain:

$$F_{es} \sim \frac{1}{4\pi \times (8.85 \times 10^{-12}) \times 3.1} \times \frac{(\varepsilon_0\varepsilon_{\text{oil}}ER^3)^2}{s^4} \sim 10^{-7} \text{ to } 10^{-6} \text{ N}$$

This force is of the same order as F_E and is sufficient to overcome the stabilising oil film between adjacent droplets. As the droplets approach within several diameters, electrohydrodynamic (EHD) flow driven by tangential electric stresses at the droplet surface becomes even more pronounced, further accelerating drainage of the intervening oil film.

(5) Summary of force balance and voltage/oil-volume dependence

The order-of-magnitude estimates are compiled in **Supplementary Table S1** (see below). The analysis leads to the following conclusions:

At low voltages (5–8 kV): $Ca_E < 1$; electric stress is insufficient to overcome surface tension; oil-film contraction is weak; droplet deformation is limited; coalescence is incomplete.

At optimal voltage (12 kV): $Ca_E \gg 1$; Maxwell stress dominates, driving strong oil-film contraction and droplet stretching; electrostatic attraction promotes droplet approach; complete coalescence is achieved.

At optimal oil volume (60 μL): film thickness $h \approx 122$ μm provides sufficient deformability while maintaining a high electric field at the oil surface; viscous dissipation is minimal.

At excessive oil volume (80 μL): the increased film thickness reduces the electric field at the oil–air interface ($E \propto 1/h$ for a fixed geometry), weakening the Maxwell stress; simultaneously, enhanced bulk viscous dissipation during film contraction impedes droplet mobility and coalescence.

This quantitative force-balance analysis substantiates our proposed mechanism and fully explains the voltage- and oil-volume-dependent coalescence behaviour observed in Figures 4c and 4d. We have incorporated these discussions into the revised manuscript and added Supplementary Table S2 for concise reference.

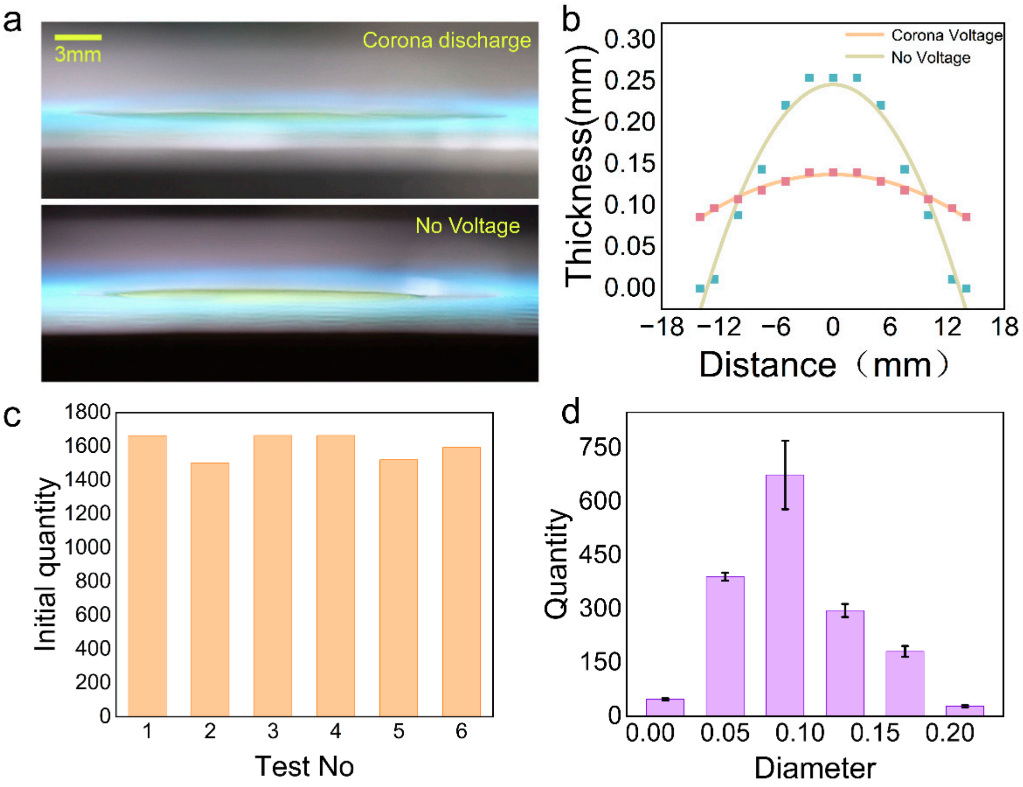

Figure S1. Quantitative characterization of the initial experimental conditions. (a) Representative images of the oil-film spreading morphology before and after corona pretreatment. (b) Oil-film thickness measured at different positions within the circular working region, showing the spatial uniformity of the film before and after corona pretreatment. (c) Initial droplet numbers obtained from six independent ultrasonic atomization experiments conducted under identical conditions, demonstrating the batch-to-batch reproducibility of the droplet population. (d) Initial droplet-size distribution determined by automated image analysis. Most droplets were distributed within the diameter range of 0.08–0.12 mm.

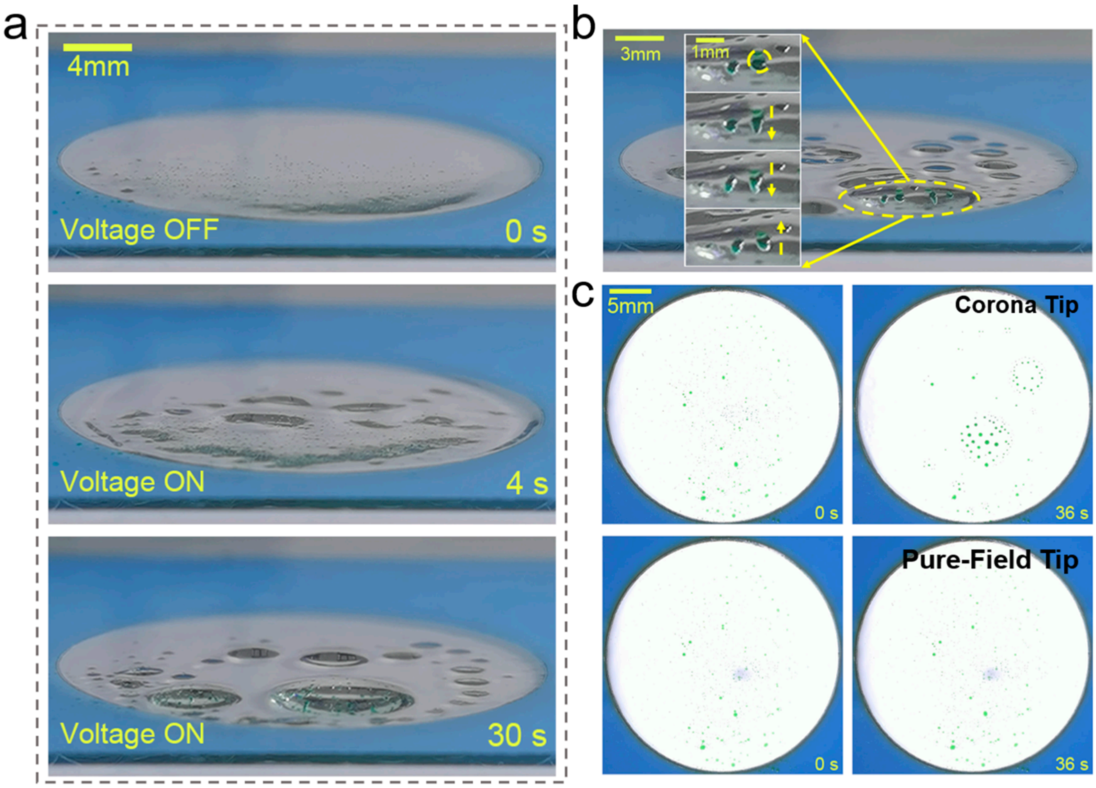

Figure S2. Experimental evidence for corona-induced oil-film contraction, droplet bouncing, and ion-transport-mediated coalescence. (a) Time-resolved oil-film contraction and inward transport of dispersed droplets under corona discharge. **(b)** Representative consecutive frames showing the approach, temporary separation, and bouncing motion of neighboring droplets. **(c)** Comparison of droplet behavior using an exposed corona tip and an insulated pure-field tip under the same applied voltage.

Table S1. Order-of-magnitude force balance analysis under typical experimental conditions (12 kV, 60 μL oil, $R = 0.1$ mm).

Force	Symbol	Expression	Estimated magnitude (12 kV)	Voltage dependence	Oil-volume dependence
Maxwell stress	F_E	$\frac{1}{2}\varepsilon_0\varepsilon_{\text{oil}}E^2 \cdot \pi R^2$	$\sim 10^{-5}$N	Strong ($\propto V^2$)	Weak (through h)
Interfacial tension	F_γ	$2\gamma R^{-1} \cdot \pi R^2 = 2\pi\gamma R$	$\sim 1.6 \times 10^{-5}$N	None	None
Viscous drag	F_μ	$\mu_{\text{oil}}Uh^{-1}\pi R^2$	$\sim 10^{-8}N$	Weak	Moderate ($\propto 1/h$)
Electrostatic attraction	F_{es}	$(4\pi\varepsilon_0\varepsilon_{\text{oil}})^{-1}p_1p_2s^{-}$	10^{-7}– 10^{-6} N	Strong ($\propto V^2$)	Moderate ($\propto s$)

Parameters used: $E \sim 10^6$ V/m, $\varepsilon_{\text{oil}} = 3.1$, $\gamma = 0.025$ N/m, $\mu_{\text{oil}} = 0.084$ Pa·s, $U \sim 0.5$ mm/s, $h = 122$ μm (60 μL oil), $s \sim 0.5$ mm.

We believe that this thorough quantitative analysis addresses the reviewer's concern and significantly strengthens the mechanistic understanding of our corona-driven coalescence platform.

Supplementary Table S2. Quantitative comparison of oil–water separation technologies.

**Technology**	**Energy consumption (kWh/m³)**	**Separation efficiency**	**Treatment time**	**Chemicals**	**Membrane fouling**	**Electrode corrosion**	**Key limitation**
**This work**	**0.3–1.6**	N/A (proof-of-concept)	~169 s	No	No	No	Current low throughput
Conventional electrocoalescence	>50	~82.5%	min–h	No	No	Yes	High energy, corrosion
Advanced E-demulsification membrane	3	91.4±3.0%	Continuous	No	Yes	No	Membrane fouling
Membrane filtration	0.02–0.03	>99%	Continuous	No	Severe	No	Membrane fouling
Centrifugation	1.0–4.0	~90%	Continuous	No	No	No	High equipment cost
Chemical demulsification	Very high	Moderate–High	Hours	Yes	No	No	Chemical cost, secondary pollution
Ultrasound	Very low (per	Moderate	s–min	No	No	No	Fails at high

	pair)						viscosity

Supporting Movies:

Movie S1. The coalescence process of dyed deionized water microdroplets deposited on the olive oil film.
Movie S2. AI-assisted detection and tracking of corona-driven droplet coalescence.
Movie S3. Macroscopic and microscopic visualization of corona-driven electro-demulsification.
Movie S4. Corona-induced oil-film contraction and charge-related droplet bouncing.
Movie S5. Control experiments distinguishing corona-ion transport from the pure electric-field effect.
